# Risk Assessment of Pharmaceutical Contaminants in Pharmaceutical Wastewater

**DOI:** 10.1155/2024/5538398

**Published:** 2024-10-28

**Authors:** Lyndon N. A. Sackey, Augustine Okobeng, Priscilla Yawa Obidieh, Flora-Marie Mpaka Ngala, Emmanuel Bentum Otoo, Jeremiah Quartey, Joseph A. Bentil, David Azanu

**Affiliations:** ^1^Department of Environmental Science, Kwame Nkrumah University of Science and Technology, Kumasi, Ghana; ^2^Department of Biochemistry and Biotechnology, Kwame Nkrumah University of Science and Technology, Kumasi, Ghana

**Keywords:** analgesic, effluent, environmental matrices, pharmaceutical contaminants, toxicity

## Abstract

The disposal of pharmaceutical wastewater has gained increasing attention due to its potential adverse effects on the environment and public health. This study aims to assess the concentration of pharmaceutical contaminants and their toxicity to aquatic organisms. A qualitative research approach was used. Composite samples were collected from the effluent storage system. Various analytical techniques, including high-performance liquid chromatography (HPLC), were employed to detect and quantify pharmaceutical compounds in wastewater samples. The results revealed various pharmaceutical compounds (ibuprofen 28 *μ*g/L, diclofenac 27.20 *μ*g/L, paracetamol 22.03 *μ*g/L, and tramadol <0.01 *μ*g/L) in the wastewater. The maximal environmental concentration (MEC) for all the detected pharmaceuticals was high; hence, the risk quotients (RQs) indicated potential toxicity (RO > 1). It further indicates that the effluent was more toxic to animals (daphnia and fish) than algae (plants). It can be concluded that pharmaceutical effluent was toxic to aquatic organisms. Therefore, there is a need to implement stringent regulatory measures to mitigate untreated pharmaceutical effluent in water bodies. Addressing pharmaceutical contaminants in wastewater is crucial to safeguarding the environment and public health in an increasing pharmaceutical use and production era.

**Summary**

• The samples have high turbidity, and the pH was acidic which could contribute to the toxicity.
• The sample analysis indicated high levels of Fe, Pb, and Cr.
• Three analgesics detected have high concentrations above the permissible limit.
• The effluent had a toxicity effect on the daphnia and fish but not the algae.

## 1. Introduction

Over the past 20 years, many research studies on emerging contaminants (ECs) have been published, covering a wide range of topics, from their occurrence in the environment to their effects on ecology. ECs are naturally occurring and man-made compounds found in the environment, such as metabolites from pharmaceuticals and personal care products [1]. Medicines will be essential to developing countries because they are needed to treat ailments. As such, they cannot be ignored. Pharmaceutical pollutants (PCs) are biologically active compounds designed to treat, prevent, or cure diseases. They are among the most concerning types of environmental contaminants (ECs) that arise from the pharmaceutical industries [2].

Borecka et al. [3] indicated an increase in the prevalence of PCs in environmental matrices. Pharmaceuticals are referred to as “pseudopersistent” contaminants because they are continuously released into the aquatic environment through a variety of pathways, such as surface runoff from urban or agricultural areas, landfill leachate, illegal disposal, and treated or untreated wastewater released from municipal, hospital, and industrial wastewater treatment plants (WWTPs) [4]. However, studies have demonstrated that certain drugs can mimic natural steroid hormone action, even at very low concentrations, resulting in similar hormonal responses [4–6]. For instance, endocrine-disrupting chemicals (EDCs) can imitate and suppress human endocrine systems, which can have serious adverse effects.

Human activities and industrial operations are the main sources of PCs [7]. Concern over these toxins' existence is growing due to the possible harm they could do to Ghana's environment and public health [8]. While pharmaceutical manufacturing facilities release effluents containing APIs during production procedures, hospitals produce wastewater containing leftover or expired medication [9, 10]. Household wastewater may contribute to the overall amount of PCs if medications and personal care items are disposed of improperly. Moreover, pharmaceutical residues pollute agricultural soils when wastewater or biosolids are used as fertilizers [11].

Pharmaceutical wastewater contains a variety of contaminants, including APIs and their metabolites, which may be detrimental to aquatic ecosystems and human health [12, 13]. Pharmaceutical and personnel care products (PPCPs) from wastewater are a potential hazard to the human health and wildlife, and their occurrence in wastewater has caught the concern of researchers recently [14]. As the pharmaceutical industry expands, so is the significance of the problem of untreated or insufficiently treated effluent discharge into the environment [15]. However, the absence of standardized, effective techniques for monitoring and assessing these PCs in pharmaceutical wastewater hinders the development of appropriate regulatory frameworks and mitigation strategies. This issue must be addressed right away by developing sensitive and trustworthy analytical techniques, putting monitoring programs in place such as risk assessment using model organisms, and developing efficient treatment processes to ensure the reduction or elimination of pharmaceutical contaminants from pharmaceutical wastewater [2]. However, the toxicity of pharmaceutical effluent to the aquatic system has not been assessed, and there is an urgent need for such research to be conducted to provide information on the level of toxicity of the effluent to aquatic organisms.

The establishment of risk assessment is very important to developing countries like Ghana where there is not advanced wastewater treatment technology to remove toxicants in the pharmaceutical effluent. The understanding of the nature of toxicity of the aquatic system as a result of the discharge of effluent such as pharmaceutical wastewater without treatment could cause environmental health problems. Even though model organisms have been used for environmental risk assessment, Sackey and Kočí [16]; Sackey, Kočí, and Gestel van [17]; Sackey, Mocová, and Kočí [18] have not been extensively conducted on pharmaceutical effluent. Therefore, there is a need to carry out such research to provide an overview of the level of toxicity to the ecological system as a result of the discharge of untreated effluent in water bodies.

This study aims to assess the concentration of pharmaceutical contaminants in wastewater and their toxicity to aquatic organisms. This research will contribute to safeguarding the integrity of ecosystems and protecting public health from potential adverse effects of pharmaceutical pollution. Also, we provide information on the status of water bodies and necessary actions that need to be taken to protect the ecosystem.

## 2. Materials and Method

### 2.1. Study Area

**Figure 1:**
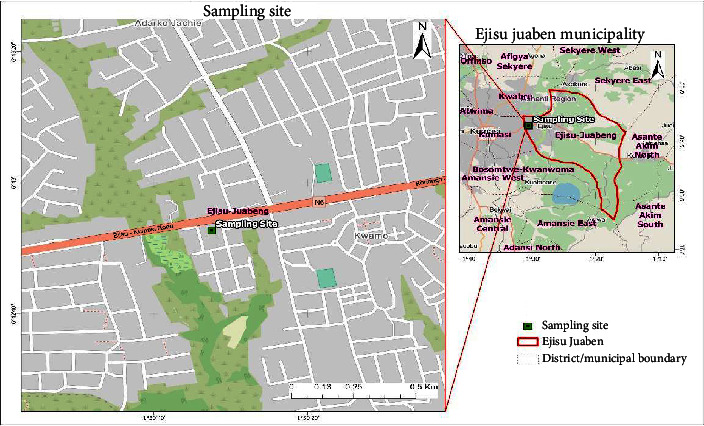
Map of the study area.

This study was carried out in the Ghanaian Ashanti region at the town of Ejisu, part of the Ejisu-Juaben municipality. With a total land area of 24,389 km^2^, the Ashanti region is the third largest of Ghana's 16 administrative regions and is situated in the country's south (Figure 1). Situated in the heart of the Ashanti region, the Municipality offers Ghana a huge chance to establish an inland port that will cater to the country's northern region. It lies between latitudes 7°9′N and 7°36′N and longitudes 1°5W and 1°39′W. It is the fifth-largest district in the Ashanti region, with a land area of approximately 1782.2 km^2^ [19].

The limits of Ejisu Municipality are shared with six other districts in the region. Sekyere East District and Kwabre East Municipal are to the northeast and northwest of the Municipal, respectively; Bosomtwe District and Asante Akim South Municipal are to the south; Asante Akim North Municipal is to the east; and Kumasi Metropolitan is to the west. The majority of the Ejisu people work in agriculture as their primary occupation. Cultivating crops such as cocoa, maize, yam, and vegetables are one aspect of farming. Still, the town's overall occupational mix includes small-scale enterprises and other professions.

### 2.2. Chemicals and Reagents

Fluka (Steinheim, Germany) provided amoxicillin trihydrate (CAS #: 267-87-780, 98% pure), while Sigma-Aldrich (Dorset, UK) provided diazepam (CAS #: 439-14-5, 98% pure) and tramadol hydrochloride (CAS #: 36,282-47-0, > 99% pure). Penicillin V, cefuroxime, ibuprofen, and acetaminophen were acquired from a Ghanaian pharmaceutical company. HPLC grade acetonitrile (CAS #: 75-05-8, > 99.9% pure) and methanol (CAS #: 67-56-1, > 99.9% pure) were provided by Merck. The stock solutions were prepared using methanol and frozen at −18°C.

### 2.3. Sampling Procedure

The samples were taken from a cesspit tank, which has been constructed for withholding effluent from production by the industry. High-density polyethylene bottle (HDPE) was used for the collection of effluent from the pharmaceutical company due to their chemical resistance and durability and ability to withstand a wide range of chemicals and is also less prone to breakage compared to glass bottles. In total, five effluent samples were collected, preserved in an ice chest with ice blocks, and transported to Kwame Nkrumah University of Science and Technology Central Laboratory for analysis.

### 2.4. Determination of pH

The pH of the samples was measured in the laboratory using an OHAUS STARTER 3100C model pH meter. The pH electrode underwent calibration. The instrument electrode was rinsed with distilled water. Each sample was assessed for pH after 50 mL was added to a beaker. The standard method procedure was used.

### 2.5. Determination of Turbidity

The 2100N brand turbidity meter was used to test the turbidity of each sample. Initially, the equipment was calibrated using formalin standards. This was accomplished by adding appropriately mixed 20 NTU, 200 NTU, and 400 NTU to a clean sample cell. Each water sample was obtained in 10 mL and placed in a Nessler. Next, the Nessler was inserted into the instrument-covered cell compartment. The value on the device was read and recorded in nephelometric turbidity units (NTU) after 5 min. The standard method procedure was used.

### 2.6. Determination of Electrical Conductivity

The OHAUS STARTER 3100C (multifunctional) conductivity meter was used to determine the samples' electrical conductivity and dissolved solids (DSs). The required calibration was performed on the instrument before it was used for all measurements. The electrode, after calibration, was placed in the sample for the measurement of conductivity and DSs. The standard method procedure was used.

### 2.7. Determination of Heavy Metals

A representative sample of pharmaceutical wastewater was collected in clean glass bottles. The wastewater was filtered using a 0.5 *μ*m filter to remove suspended solids. A known volume of the filtered wastewater sample was transferred into a digestion vessel. The volume depends on the heavy metals' expected concentration and the analytical instrument's detection limits. The appropriate digestion acids (e.g., nitric acid) were added to the sample in the digestion vessel. The acid concentration and volume were sufficient for the complete digestion of the sample.

The sample was digested using a suitable digestion apparatus, such as a hot plate, digestion block, or microwave digestion system. Recommended digestion methods and parameters for the heavy metals of interest were followed [20]. It was ensured that appropriate safety precautions were taken during digestion, such as working in a fume hood and using acid-resistant gloves, lab coats, and safety goggles. After digestion, the sample was allowed to cool, and the digested solution was transferred into a clean container.

The calibration standards for each heavy metal of interest were prepared by diluting the stock standard solutions to different concentrations. The calibrated MP-AES Agilent 42,100 was used to measure the concentrations of the heavy metals in the digested wastewater sample. The instrument's digested sample, calibration standards, and quality control samples were done, ensuring proper rinsing between measurements to avoid contamination.

The concentrations of the heavy metals in the wastewater sample were calculated based on the calibration curve and instrument response.

### 2.8. Analytical Procedure for Pharmaceutical Contaminants Determination

#### 2.8.1. Solid-Phase Extraction (SPE) and Analysis

The SPE procedure described by Azanu et al. [21] was modified and used in this study. Water samples were cleaned up and concentrated on Oasis HLB (hydrophilic–lipophilic balance, 200 mg sorbent, 30 m, 6 cm^3^) cartridge supplied by Water Oasis (SPE) (Massachusetts, USA), and 2 mL MeOH, after that 2 mL distilled water, was used to condition the SPE cartridge. At a flow rate of 1.5 mL/min, 500 mL of water samples were put into SPE columns. Dried SPE columns were washed with 3 mL of 5% MeOH. After permitting the sorbent under a vacuum to dry for a few minutes, the antibiotics were eluted with 3 mL MeOH at a flow rate of about 1 mL min^−1^. Eluates were dried at 30°C with a moderate nitrogen flow before being reconstituted in 1 mL 1% MeOH and injected into brown flat-cap HPLC vials for analysis.

#### 2.8.2. High-Performance Liquid Chromatography (HPLC) Analysis for Four Analgesics: Ibuprofen, Tramadol, Diclofenac, and Paracetamol

The method for analyzing the analgesics was developed using a Cecil-Adept Binary Pump HPLC and a Wave Quest CE4300 UV/Vis Detector (Cambridge, UK). Zorbax (C18, 4.6 × 250 mm, 5 *μ*m, Agilent Technologies Inc., Palo Alto, CA, USA) was used for the chromatographic separation of diclofenac; SunFire (C18, 4.6 × 150 mm, 5 *μ*m, Waters, Milford, MA, USA) was used for the separation of tramadol, ibuprofen, and paracetamol; this was done at 30°C after a guard column (SunFire, C18, 4.6 × 10 mm, 5 *μ*m, Waters, Milford, MA, USA) was used. The mobile phase was pumped at 0.8 mL/min and included 40:60 (v/v) methanol: 0.1 M sodium acetate buffer (pH = 4). For analysis, a 10 *μ*L sample was put into the HPLC.

### 2.9. Risk Assessment

According to EMEA guidelines, the risk quotient (RQ) of each pharmaceutical contaminant was calculated as the ratio of the contaminant's maximum measured environmental concentration (MEC) to its projected noneffect concentration (PNEC). Therefore, the assessment factor (AF) was set to 5, which reduced the uncertainty of results to a certain extent. PNEC was calculated using the following equation [22]:

(1) $$\begin{array}{c} \text{PNEC}=\frac{{\text{HC}}_{5}}{\text{AF}}. \end{array}$$

To support all trophic levels, RQ for fish, algae, and daphnia was established. RQ value was calculated using the following equation (Tian et al., 2020):

(2) $$\begin{array}{c} \text{RQ}=\frac{\text{MEC}}{\text{PNEC}}. \end{array}$$

Using the ecological structure–activity relationships (ECOSAR) model, PNEC data were acquired for this investigation. To account for the inherent uncertainty of measured concentrations, the AF was multiplied by the PNEC values obtained from the model. The AF values of 100 that Leung et al. [23] utilized were applied to this investigation. A criterion for risk rating is set at RQ < 0.1, signifying little danger to aquatic species, 0.1 ≤ RQ ≥ 1, signifying medium risk, and RQ > 1, signifying severe risk.

### 2.10. Data Analysis

The Pearson's correlation coefficient test was used to determine the relationship between the concentrations of each pharmaceutical.

## 3. Results

### 3.1. Physiochemical Parameters

The physiochemical test was performed based on six parameters for the samples collected, and the results are shown in Table 1.

### 3.2. Determination of Heavy Metals

Cadmium, chromium, iron, and lead were the heavy metal of interest whose concentrations in mg/L in the samples were determined, and the results are shown in Table 2.

### 3.3. Determination of Pharmaceutical Contaminants Using HPLC

**Figure 2:**
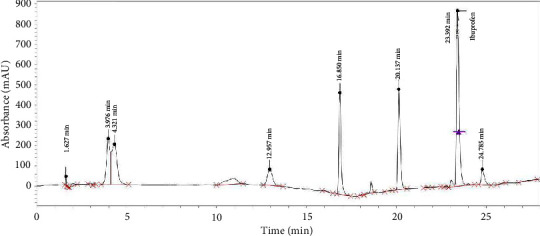
Graphical display of absorbance versus retention time for various ibuprofen and their respective peaks from HPLC analysis.

Figure 2 represents the chromatograph from the HPLC analysis showing the analgesics present and their peaks. The horizontal axis represents the retention time, while the vertical axis shows the absorbance. Paracetamol has the highest peak height of 169,914.60 and an area of 2,041,425.60 which is commensurate with its high concentration detected and tramadol with the lowest height of 16,906.50 and an area of 114,273.60 which indicate that its concentration is below detection limit.

Table 3 shows that ibuprofen had the highest peak number of 13 with the highest retention time of 23.464 min. and the highest final concentration of 28.67 *μ*g/L. It had the second lowest area and height compared to the tramadol, with the lowest area, height, and final concentration of 114,273.6, 16,906.5, and 0, respectively. It also recorded 13.981 min. as retention time and 9 as its peak value. Diclofenac comes second in final concentration, retention time, and peak value, corresponding to 27.20, 23.064 mins, and 12, respectively. Its area and height values were 1,032,860.5 and 138,373.3, respectively. On the other hand, paracetamol had the lowest peak value and retention time at 5 and 3.745, respectively. It also recorded 22.03 *μ*g/L as the final concentration and 2,041,425.6 and 169,914.6, respectively.

### 3.4. Risk Assessment

Ibuprofen recorded the highest MEC value of 28.679 *μ*g/L, resulting in the highest RQ of 0.6972 in algae, 1.0298 in daphnids, and 0.6900 in fish. Paracetamol recorded 22.035 *μ*g/L as the MEC value, which produced an RQ of 0.0266 in algae, 0.0102 in daphnids, and 0.0049 in fish. Tramadol had the lowest environmental concentration of 0.00 *μ*g/L indicating 0 value at all trophic levels. Diclofenac recorded the second-highest MEC value of 27.202 *μ*g/L, giving RQ values of 0.6568, 1.0562, and 0.7224 in algae, daphnids, and fish, respectively (Table 4).

The value obtained from Pearson's correlation coefficient (*r*) was 0.46 (Table 5). This indicates a moderate positive correlation. This means that when the concentration of one pharmaceutical increases the concentration of the other pharmaceuticals increases as well but not too strongly.

## 4. Discussion

### 4.1. Physiochemical Parameters

One of the most important factors affecting the quality of water and soil is pH. While some aquatic species can survive in water with a pH outside of this range, most prefer a pH range of 6.5–9.0. pH values in aquatic environments. Fish should have a pH between 6.5 and 9.0. In environments that are not ideal, organisms may suffer stress or even perish. This study's average pH value was 5.15, which is slightly acidic and lower than the 6.5 minimum GSB/WHO recommendation level. The release of acidic compounds or byproducts from pharmaceutical production operations may be the cause of low pH in pharmaceutical wastewater [24]. In general, wastewater with pH extremes is undesirable since they may jeopardize aquatic life's ability to survive [24].

The samples' average turbidity values were higher than the suggested 5.0 NTU GSB/WHO standard. Particles from manufacturing processes, chemicals, or medicine residues are examples of suspended solids that could be the cause of this high turbidity value [24]. Water with a high turbidity level can harm the ecosystem in a number of ways. It damages algae and aquatic plants by reducing the quantity of light that can reach the water [25]. Significant increases in turbidity have the potential to disrupt the ecosystem and impede the growth of both plants and animals. High amounts of particulate matter will change light penetration when sunlight is obstructed, causing narrow lakes and bays to fill in more quickly and suffocate benthic habitats. This affects the eggs of aquatic species as well. Additionally, less photosynthesis will occur, which lowers the production of marine plants in the waterbed, such as bay grasses and seaweed. Furthermore, cloudiness detracts from the visual value of water features, which can be detrimental to tourism and leisure.

All of the study's average TDS levels fell below the less than 1000 mg/L GSA/WHO acceptable guideline. The colloidal form and dissolved species were represented by the total solid content in the waste effluent. The content collision of these colloidal particles was the main cause of the variation in the total solid value and, subsequently, the DS value. The pH of the effluent also affects the aggregation process's collision rate [26]. Excessive TDS concentrations can degrade water quality and render it unfit for irrigation or consumption. Additionally, it can damage aquatic life by changing their osmoregulation, leading to stress or even death [26].

All of the average electrical conductivity values found in this study fell below the 1000 µS/cm GSB/WHO threshold. Otoo et al. [24] suggest that the low concentration of ions or dissolved salt in the water may be the cause of the low electrical conductivity observed in pharmaceutical effluent. The average conductivity values that were recorded were less than the values that Nkansah, Boadi, and Badu [27] reported, which ranged from 46 to 282 µS/cm. The electrical conductivity of water is also influenced by temperature. Its conductivity likewise increases as the temperature rises. By upsetting the equilibrium of ions and nutrients in the water, it can have an impact on aquatic ecosystems, resulting in detrimental algal blooms and lowered oxygen levels. This denotes the release of pharmacological samples into the environment; neither land creatures nor the aquatic system will be harmed by the conductivity.

### 4.2. Heavy Metals

Pharmaceutical wastewater has a lead value of 0.06 mg/L, which is more than the 0.01 mg/L WHO recommended standard level for lead [28]. Lead reagents (lead acetate, lead nitrate, and lead oxide), lead-based catalysts (lead tetra acetate and lead oxide), and lead-containing materials (lead-base pigments or dyes) are all responsible for the presence of lead in pharmaceutical wastewater [24]. Due to lead's toxicity and possible danger to human health, high amounts of lead in wastewater can have a negative impact on the environment. Lead is a heavy metal. If improperly managed, it can also build up in aquatic environments and potentially make its way into the food chain [24]. Because lead is a potent neurotoxin, exposure to it can harm the brain. Lead exposure can also cause harm to other soft tissues and organs, obstruct blood coagulation, and possibly result in death. Lead exposure can have negative health impacts on both adults and children.

Pharmaceutical wastewater has a chromium concentration of 0.06 mg/L, which is more than the 0.05 mg/L WHO-recommended standard limit for total chromium [28, 29]. The reason for the presence of chromium is that the pharmaceutical business uses chromium-containing materials to make their equipment, including chromium oxide, chromium acetate, and chromium chloride [30]. Using catalysts based on chromium or chromium salt is an additional approach that is feasible. Additionally, corrosion of chromium-plated pipes or equipment might allow chromium to infiltrate wastewater [24]. Because chromium is a heavy metal that, depending on its form and concentration, can have both toxic and carcinogenic effects, concentrations above allowable limits can pollute the environment and pose health hazards.

Pharmaceutical effluent with an iron concentration of 1.45 mg/l has a comparatively high iron content. Iron can cause water to turn reddish or brownish when present in elevated concentrations, which is likely the cause of the reddish color shift that was seen following filtering [21]. Elevated iron levels can cause esthetic problems such as discoloration and unwanted taste or color, even though iron itself is generally not hazardous to human health in concentrations commonly seen in water [21]. In addition, sedimentation and scale buildup in equipment, and pipes can result from too much iron in the water. Iron in pharmaceutical wastewater is thought to be present in iron-containing compounds or reagents such as iron (III) oxide, iron (III) chloride, and iron (II) sulfate. Because of the high amounts of iron in the sample, releasing it into the environment untreated may have negative effects on the aquatic system.

### 4.3. HPLC Analysis

The analysis revealed the presence of ibuprofen, paracetamol, and diclofenac. Only tramadol was absent. Ibuprofen, paracetamol, and diclofenac have all been found to have ecotoxic effects on aquatic organisms. Pharmaceuticals can bioaccumulate in the tissues of aquatic organisms. This means that as smaller organisms ingest these drugs, they can be passed up the food chain, potentially reaching concentrations harmful to predators, including humans, if they consume contaminated fish or seafood. The presence of pharmaceuticals in wastewater can also contribute to the development of antibiotic resistance in bacteria [31]. Releasing analgesics like diclofenac into the environment can exert selective pressure on bacteria, leading to resistant strains [24, 32]. This poses a significant public health risk.

### 4.4. Risk Assessment

The MEC of analgesics in pharmaceutical wastewater collected from pharmaceutical effluent was used to calculate the RQ. PNEC values and RQs for each analgesic are shown in Table 4.

The RQ values for paracetamol were 0.02, 0.01, and 0.01 for exposure to algae, daphnids, and fish, respectively. All the values were lower than 0.1 indicating low toxicity risk. The RQ values for diclofenac were 0.65, 1.06, and 0.72 for exposure to algae, daphnids, and fish, respectively. However, studies have shown that RQ > 1 for diclofenac can lead to endocrine disruption by acting on the prostaglandin pathway in rodents and human cells due to hindrances in the prostaglandin synthesis [24]. The RQ values for ibuprofen were 0.70, 1.03, and 0.70 for exposure to algae, daphnids, and fish, respectively. Ibuprofen poses a high risk to fish with an RQ > 1, which can impact fish reproduction by male fish feminization [24]. Male fish feminization can reduce the fish population and hence have an economic impact. Since tramadol did not show any peak in our studies, there were no RQ values, hence no impact on the species.

## 5. Conclusion

From the study, turbidity, TDS, pH, salinity, and temperature were not within the GSA/WHO permissible limits. The electrical conductivity was within the acceptable limit of GSA/WHO. The low pH and high turbidity could corrosively affect the aquatic organisms within the environment. The sample analysis revealed the presence of several heavy metals, including iron, lead, and chromium with concentrations of 1.45 > 0.06 > 0.05 mg/L, respectively. Interestingly, cadmium was under detection (<0.01 mg/L). The study's findings confirmed the presence of 3 analgesics: paracetamol, diclofenac, and ibuprofen, while tramadol was not detected. The MEC for all the detected pharmaceuticals was high; hence, the RQs indicated potential toxicity (RO > 1). It further indicated that the effluent was more toxic to animals (daphnia and fish) than algae (plants). The existence of pharmaceutical contaminants in the environment is not solely a worry for the environment but also a human health threat because it can impact groundwater and be uptake by vegetables. The presence of lead, iron, and chromium in the sample suggests the need for closer monitoring and assessment of potential risk. Additionally, continued monitoring is essential to ensure that appropriate measures are taken to mitigate any adverse effects on human health and the environment. The high concentrations of pharmaceuticals in the environment can be reduced by adopting proper disposal methods. The result can help in policy and decision-making in the protection of aquatic systems [33].

## Figures and Tables

**Table 1:** Values obtained from the physiochemical test.

Parameter	Unit	Measurement
pH	—	5.15
Electrical conductivity	*μ*s/cm	463.00
Total dissolved solids	mg/L	231.00
Turbidity	NTU	31.00
Salinity	psu	0.22
Temperature	°C	27.1

**Table 2:** Values obtained from the four heavy metal analyses.

Heavy metal	Measurement (mg/L)
Pb (405.781 nm)	0.06
Cd (228.802 nm)	−0.04
Fe (371.993 nm)	1.45
Cr (425.433 nm)	0.05

*Note:* Pd = lead, Cd = cadmium, Fe = iron, Cr = chromium.

**Table 3:** Results obtained from HPLC test for 4 analgesics of study.

Peak#	Rt (min)	Component name	Area	Height	Bl	Concentration (*μ*g/L)
5	3.74	Paracetamol	2,041,425.60	169,914.60	BB	22.03
9	13.98	Tramadol	114,273.60	16,906.50	BB	< 0.01
12	23.06	Diclofenac	1,032,860.50	128,373.30	BV	27.20
13	23.46	Ibuprofen	263,300.30	33,247.70	VB	28.67

*Note:* # = number.

Abbreviations: BB = baseline to baseline, BL = baseline, RT =  retention time, VB = valley to baselines.

**Table 4:** Risk assessment of aquatic organisms.

Analgesics	MEC (*μ*g/L)	PNEC algae (*μ*g/L)	RQ algae	PNEC daphnid (*μ*g/L)	RQ daphnid	PNEC fish (*μ*g/L)	RQ fish
IBU	28.68	41.13	0.70	27.85	1.03	41.56	0.69
PARA	22.04	829.66	0.03	2157.16	0.01	4457.75	0.00
TRA	0.00	18.24	0.00	16.63	0.00	26.68	0.00
DIC	27.20	41.41	0.66	25.75	1.06	37.65	0.72

**Table 5:** Results obtained from the Pearson's correlation test between 4 analgesics.

Total	*X*	*Y*	*x* = *X* − $\bar{x}$	*y* = Y − $\bar{y}$	*x* ^2^	*y* ^2^	Xy
	PARA	22.04	−1.50	2.56	2.25	6.53	−3.83
	TRA	0.00	−0.50	−19.48	0.25	379.43	9.74
	DIC	27.20	0.50	7.72	0.25	59.64	3.86
	IBU	28.68	1.50	9.20	2.25	84.64	13.80
∑ =	2.5	19.48	0.00	0.00	5.00	530.25	23.57

## Data Availability

Data are available upon request.
